# A Case of Bilateral Auricular Dystonia With Both Sensory Trick and Reverse Sensory Trick Successfully Treated With Botulinum Toxin Therapy

**DOI:** 10.7759/cureus.78896

**Published:** 2025-02-12

**Authors:** Chiaki Takahashi, Takashi Asahi, Isao Matsushita

**Affiliations:** 1 Department of Rehabilitation Medicine, Kanazawa Medical University, Uchinada, JPN; 2 Department of Neurosurgery, Kanazawa Neurosurgical Hospital, Nonoichi, JPN

**Keywords:** auricular dystonia, botulinum injection, moving ear syndrome, reverse sensory trick, sensory trick

## Abstract

The patient was a 39-year-old male who presented to our hospital with a 15-year history of sustained involuntary movements of both auricles. A phenomenon suspected to be a sensory trick was observed, in which pressing on the frontal region above the eyebrows ceased the involuntary movements and was accompanied by compensatory contraction of the frontalis muscle. It was considered that this sensory trick, induced by pressure stimulation, led to the simultaneous occurrence of two distinct behaviors: the improvement of auricular dystonia and the emergence of involuntary contraction of the frontalis muscle. Botulinum toxin A was administered to both the auricular muscles and the frontalis muscle, resulting in favorable control of the symptoms.

Reports of bilateral auricular dystonia are exceedingly rare. Moreover, the concurrent appearance of two types of sensory tricks - one alleviating dystonia and the other exacerbating it - is also uncommon. When administering botulinum toxin A injection in such cases, it may be beneficial to consider not only for the primary dystonia but also for the sites where reverse sensory tricks manifest, potentially achieving better outcomes.

## Introduction

Dystonia, as a type of involuntary movement, involves intermittent or sustained involuntary muscle contractions that can occur in various localized regions of the body. In the head and neck region, rare cases of dystonia affecting the auricles, known as "auricular dystonia" or "moving ear syndrome" and other designations, have been reported [1]. In such cases, the anterior, superior, and posterior auricular muscles, normally incapable of voluntary movement in humans, are observed to contract involuntarily [2]. In any form of dystonia, phenomena known as "sensory trick" or "geste antagoniste" may be observed [3]. A sensory trick is a phenomenon in which involuntary movements caused by dystonia are temporarily alleviated through specific actions such as sensory stimulation, postural adjustments, or certain movements [2]. There is a report that investigated 164 cases of cervical dystonia, revealing that 89.6% of the cases utilized sensory tricks, with approximately 80% of those demonstrating a reduction in involuntary movements [4]. Furthermore, it has been reported that certain types of stimuli not only alleviate dystonia symptoms but, in rare cases, may also exacerbate them. This phenomenon is referred to as a "reverse sensory trick" [5,6]. In this report, we present a rare bilateral case of auricular dystonia with both sensory trick and reverse one, successfully treated with botulinum toxin A.

## Case presentation

**Figure 1:**
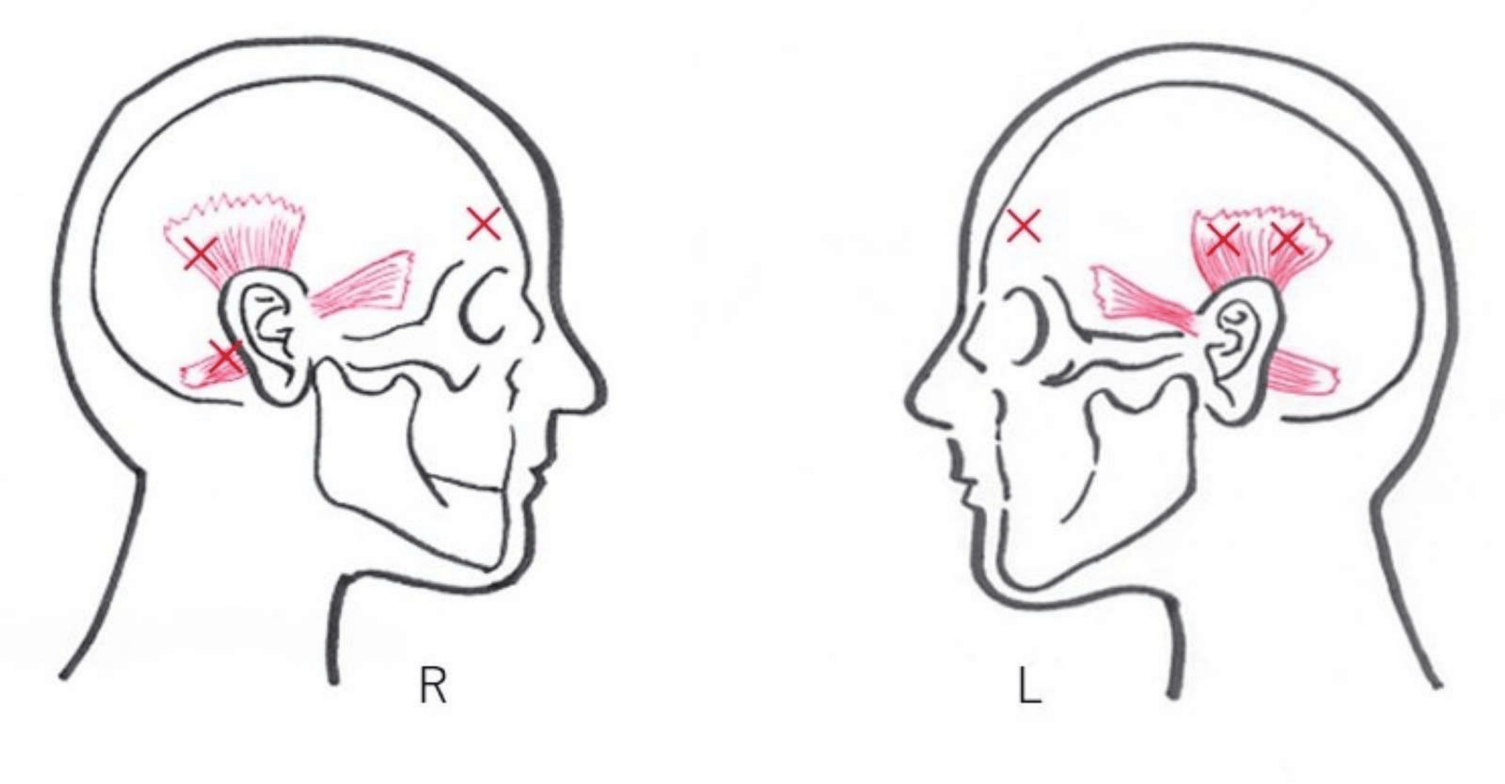
Injection sites The injection sites of botulinum toxin A on the left and right sides are indicated. Original figure created by Chiaki Takahashi.

A 39-year-old male office worker presented with a 15-year history of involuntary movements of both auricles, which disrupted his concentration at work. He also stated that the involuntary movements ceased during sleep. He had no history of head trauma, psychiatric disorders, or other significant medical problems. On examination, semi-rhythmic, sustained involuntary movements of both auricles were observed: the right auricle was pulled backward and the left auricle upward. Palpation revealed persistent involuntary contraction of the right superior and posterior auricular muscles and the left superior auricular muscle. Pressing stimulations of the frontalis muscles above the eyebrows bilaterally alleviated the auricular movements but induced new dystonic contractions of the frontalis muscles, causing upward eyebrow movement. A video of the more pronounced right-sided dystonia is shown (Video 1). The patient exhibited no other cranial nerve abnormalities, and brain MRI findings were normal, and electromyography was not performed. A diagnosis of auricular dystonia was made, and initial treatment with zolpidem (10 mg) administered but showed no effect. Therefore, botulinum toxin A injection was administered, with a total dose of 40 units: 25 units on the right (10 units to the superior, 10 units to the posterior auricular, five units to the frontalis muscle) and 15 units on the left (10 units to the superior auricular, and five units to the frontalis muscle) (Figure 1).

**Video 1:** Auricular dystonia A video demonstrating the higher amplitude right auricular dystonia is presented. Semi-rhythmic and sustained involuntary movement is observed. At 0:11, compression above the eyebrow initiates contraction of the frontalis muscle and temporalis muscle centered around the compression site. At 0:24, the auricular dystonia is alleviated during this maneuver.

Initially, we had planned to administer botulinum toxin only to the surrounding muscles in the next session. The patient was anxious that the contraction of the frontalis muscle might become more pronounced during the treatment process. Additionally, as confirmed in the video, involuntary contraction can be observed even after the fingers are released. Therefore, considering that the contraction of the frontalis muscle may worsen and become more pronounced due to factors such as stress or fatigue in the future, we decided to proceed with the administration. Two weeks post-treatment, the auricular dystonia and contraction of frontalis muscle had nearly disappeared (Video 2), and this effect persisted for three months. Subsequent regular botulinum toxin A treatments, administered every three months, have maintained good quality of life (QOL) for the patient.

**Video 2:** Auricular dystonia after botulinum injection Two weeks after botulinum toxin administration.

## Discussion

This case highlights the successful management of a rare bilateral auricular dystonia, and its associated sensory trick and reverse one using botulinum toxin A.

According to Ponglikitmongkol et al., 27 cases of involuntary movements of the ear have been reported in the literature, though this includes cases of ear tics and those that remain unclassified [2]. Cases that are likely to be classified as auricular dystonia are an extremely rare condition, with around 10 cases of them. Oral medications have been reported to include analgesics and antiepileptic drugs such as pregabalin [1] and clonazepam [7], as well as antidepressants; however, their efficacy varies depending on the case. At the same time, the use of botulinum toxin injections targeting the auricular muscles was first reported by Srirompotong et al. in 2007 [8]. To date, only four cases have been documented, three cases showed improvement with botulinum toxin [7-9]. Botulinum toxin blocks neuromuscular transmission through decreased acetylcholine release, which finally leads to muscle atrophy and weakness. Regarding botulinum toxin A, systemic side effects are rarely observed; however, it causes a reduction in contraction at the local muscle unit level [10]. Therefore, in cases of cranio-cervical dystonia, where the causative muscles are small, precise injection is required. For example, in this case, an excessive dose injected into the frontalis muscle may lead to transient ptosis, while an injection into the auricular muscle that accidentally affects the underlying temporalis muscle could result in transient masticatory dysfunction. To avoid such side effects, injections guided by needle electromyography stimulation or using ultrasound guidance are useful [10]. While there has been a reported case in which botulinum toxin therapy was insufficiently effective, and pain associated with dystonia was also present. Therefore, a pallidothalamic tractotomy was performed, successfully controlling both symptoms [7].

Focal dystonias without identifiable genetic or structural lesions are classified as idiopathic (i.e., blepharospasm or some cervical dystonia), and our case is considered to correspond to isolated focal dystonia. It has been reported that dysfunction due to altered connectivity between the cerebellum and the somatosensory cortex may be involved [11]. Therefore, in patients with dystonia, the inhibitory control that is normally exerted by the cerebellum on the motor and somatomotor cortex via the thalamus is absent, leading to excessive motor activity due to cortical hyperexcitability. Additionally, with regard to cases in which sensory trick is present, it has been reported that functional connectivity in sensorimotor, visual, and executive cortical domains have decreased, and furthermore, cerebellar activity is increased in response to triggering stimuli [12,13]. Thus, one possible mechanism of sensory trick is that sensory input modulates the connectivity of higher order cortical regions. Hok et al. [14] reported that in cases who responded to botulinum toxin A, the functional connectivity between the cerebellum and cortical domains decreased in proportion to the treatment effect [14]. Therefore, sensory trick and botulinum toxin A treatment may share similar mechanisms in terms of their effectiveness. Additionally, it has been reported that cases exhibiting sensory trick tend to respond well to botulinum toxin therapy and require lower effective doses [15].

Sensory trick is often identified by patients themselves as a way to suppress their involuntary movements. It has been documented that various types of stimuli, including tactile, visual, auditory, and thermal stimuli, can effectively serve as sensory tricks [3]. In our case, the phenomenon, that involuntary movements of both auricles can be inhibited by compressing the frontalis muscle, was also self-reported. Similarly with the case, Jabbour et al. reported a case in which contraction of the frontalis muscle by raising the eyebrows led to the cessation of auricular dystonia [1]. In addition, in cases where sensory tricks have been applied to treatment, it has been reported that the hanger reflex alleviates cervical dystonia and that its effectiveness improves with repeated application [16]. Interestingly, sensory trick can sometimes exacerbate or induce other dystonic symptoms, a phenomenon that Wider et al. proposed as "reverse sensory trick" [5]. A study investigating cases of cervical dystonia reported that dystonic muscle activity was elicited by tactile stimulation of the occipital or cervical region in 12.8% of cases [6]. Despite being a phenomenon observed in such a proportion of cases, there are very few investigative reports, with only two reports identified in the literature to the best of our review. It has been reported that cases with reverse sensory trick tend to overload sensory stimulus information, compared to cases with typical sensory trick, and the cases of cervical dystonia reported by Asmus et al. were presumed to be due to a dependence on visual information [6]. The simultaneous occurrence of both phenomena represents a highly complex issue and suggests a paradoxical fact. We hypothesize two possibilities: first, the presence of visual information indicating the fingers approaching during stimulation may have been perceived as excessive excitation, potentially affecting direct inter-cortical connectivity; second, the involuntary contractions observed directly beneath the site of pressure stimulation may suggest the involvement of a peripheral mechanism, similar to that seen in conditions such as hemifacial spasm. In our case, it is suggested that an undetectable dystonia in the frontalis muscle, not apparent upon visual inspection, may have been latent and triggered by compressive stimulation as a reverse sensory trick.

Regarding this case, if we had been able to evaluate electromyographic changes in the auricular and frontal muscles before and after botulinum toxin treatment, it might have provided clearer evidence of involuntary movement control and allowed for the estimation of the mechanisms underlying the emergence of sensory trick and reverse sensory trick. This is considered a limitation.

## Conclusions

In this case, we report a patient with rare bilateral auricular dystonia, for which sensory trick was effective, who also developed a novel dystonia of the frontalis muscle due to a reverse sensory trick. Both conditions were treated with botulinum toxin therapy, resulting in successful symptom control. Botulinum toxin therapy is highly likely to be effective and is recommended, particularly for cases of cranio-cervical dystonia, including auricular dystonia, in which sensory trick is effective. In cases like this, where a new dystonia emerges locally as a reverse sensory trick, it may also be appropriate to consider botulinum toxin therapy targeting the newly emerged dystonia to control the sensory overloading. In this case, the novel dystonia induced by the reverse sensory trick was fortunately well controlled with botulinum toxin therapy, as it was triggered directly beneath the stimulation site. However, if the stimulation site and the induced dystonic region are distant from each other, optimizing the injection strategy would present a challenge.
